# Interoperability between phenotypes in research and healthcare terminologies—Investigating partial mappings between HPO and SNOMED CT

**DOI:** 10.1186/s13326-016-0047-3

**Published:** 2016-02-09

**Authors:** Ferdinand Dhombres, Olivier Bodenreider

**Affiliations:** National Library of Medicine, National Institutes of Health, Bethesda, MD USA

**Keywords:** Partial mapping, Human phenotype, Ontology, Standard terminologies, Interoperability

## Abstract

**Background:**

Identifying partial mappings between two terminologies is of special importance when one terminology is finer-grained than the other, as is the case for the Human Phenotype Ontology (HPO), mainly used for research purposes, and SNOMED CT, mainly used in healthcare.

**Objectives:**

To investigate and contrast lexical and logical approaches to deriving partial mappings between HPO and SNOMED CT.

**Methods:**

1) Lexical approach—We identify modifiers in HPO terms and attempt to map demodified terms to SNOMED CT through UMLS; 2) Logical approach—We leverage subsumption relations in HPO to infer partial mappings to SNOMED CT; 3) Comparison—We analyze the specific contribution of each approach and evaluate the quality of the partial mappings through manual review.

**Results:**

There are 7358 HPO concepts with no complete mapping to SNOMED CT. We identified partial mappings lexically for 33 % of them and logically for 82 %. We identified partial mappings both lexically and logically for 27 %. The clinical relevance of the partial mappings (for a cohort selection use case) is 49 % for lexical mappings and 67 % for logical mappings.

**Conclusions:**

Through complete and partial mappings, 92 % of the 10,454 HPO concepts can be mapped to SNOMED CT (30 % complete and 62 % partial). Equivalence mappings between HPO and SNOMED CT allow for interoperability between data described using these two systems. However, due to differences in focus and granularity, equivalence is only possible for 30 % of HPO classes. In the remaining cases, partial mappings provide a next-best approach for traversing between the two systems. Both lexical and logical mapping techniques produce mappings that cannot be generated by the other technique, suggesting that the two techniques are complementary to each other. Finally, this work demonstrates interesting properties (both lexical and logical) of HPO and SNOMED CT and illustrates some limitations of mapping through UMLS.

## Introduction

In parallel to the deep sequencing effort enabled by Next Generation Sequencing technologies, a need for deep phenotyping has emerged [1]. Clinical phenotypes can be recorded in reference to multiple terminologies, including the Human Phenotype Ontology (HPO), mainly used for research purposes, and the Standardized Nomenclature of Medicine Clinical Terms (SNOMED CT), mainly used in healthcare. The interoperability of phenotypes between datasets (including electronic health record data) annotated with different terminologies is critical to translational research [2] and rests on the interoperability between the corresponding terminologies. For example, electronic health record (EHR) data coded with SNOMED CT are increasingly used as a resource for cohort selection (e.g., for selecting patients exhibiting a specific phenotype defined in reference to HPO). In this case, a mapping between SNOMED CT and HPO is key to bridging between datasets annotated to different terminologies.

The interoperability between HPO and SNOMED CT can be addressed in several complementary ways, through complete or partial mappings. Moreover, these two types of mappings can be obtained lexically (through the lexical properties of phenotype names) or logically (through the logical definitions and the hierarchical arrangement of phenotype concepts).

*Complete lexical mappings* identify exact and normalized matches between existing (“pre-coordinated”) terms in HPO and SNOMED CT and denote equivalent relations between the corresponding concepts. In previous work, we showed that only 30 % of HPO concepts could map to pre-coordinated SNOMED CT concepts [3]. For example, *Multicystic dysplastic kidney* [HP:0000003] maps to *Multicystic renal dysplasia* [SCTID:204962002] (through synonymy).

*Complete logical mappings*. Since both HPO and SNOMED CT are developed using description logics, it is be possible to compare the logical definitions of phenotype concepts between the two terminologies. However, given the differences in modeling choices in HPO and SNOMED CT, few matches would be expected. Instead, in previous work, we analyzed the logical definitions of existing phenotype concepts in SNOMED CT and created patterns (“post-coordinated expressions”) from these definitions that could be applied to HPO phenotypes not represented in SNOMED CT as pre-coordinated concepts. Through this approach, 1617 additional mappings could be identified between HPO and SNOMED CT [4]. For example, *Aplastic clavicle* [HP:0006660] would be equivalent to the following post-coordinated expression in SNOMED CT: ‘*Disease* and (Role group some ((Associated morphology some *Hypoplasia*) and (Occurrence some *Congenital*) and (Finding site some *Clavicle*)))’.

*Partial lexical mappings* identify matches similar to complete lexical mappings, but allow some words of the HPO terms to be omitted in the mapping to SNOMED CT. Such mappings denote subsumption (subclass) relations between the more specific HPO concept and the more general SNOMED CT concept mapped to. For example, *Bilateral renal atrophy* [HP:0012586] maps to the more general concept *Atrophy of kidney* [SCTID:197659005] (ignoring the modifier *bilateral*). Leveraging the compositional features of HPO terms for mapping purposes had already been suggested by [5].

*Partial logical mappings* identify a subclass relation between one fine-grained HPO concept and a more general SNOMED CT concept, when an ancestor of the source HPO concept is equivalent to some SNOMED CT concept. For example, the concept *Oral cleft* [HP:0000202] is in subclass relation to *Abnormality of the mouth* [HP:0000153] in HPO, and *Abnormality of the mouth* is equivalent to the SNOMED CT concept *Congenital anomaly of mouth* (*disorder*) [SCTID:128334002] through a complete lexical mapping. Therefore, a partial logical mapping (denoting a subClassOf relationship) can be inferred between *Oral cleft* [HP:0000202] and *Congenital anomaly of mouth* (*disorder*) [SCTID:128334002].

The objective of this paper is to investigate and contrast lexical (based on lexico-syntactic properties of clinical phenotype terms) and logical (based on subsumption relations between phenotype concepts) approaches to deriving partial mappings between HPO and SNOMED CT.

## Background

In this section, we introduce the resources used in this investigation (HPO, SNOMED CT and the UMLS). We briefly review related work on partial mappings and present the specific contribution of our work.

### Resources

*HPO*. The Human Phenotype Ontology (HPO) is an ontology of phenotypic abnormalities developed collaboratively and used for the annotation of databases such as OMIM (Online Mendelian inheritance in Man) and Orphanet (knowledge base about rare diseases) [6]. The version of HPO used in this investigation is the (stable) OWL version downloaded on January 21, 2015 (build #1337) from the HPO website (http://www.human-phenotype-ontology.org/). It contains 10,589 classes (concepts) and 16,807 names (terms) for phenotypes, including 6218 exact synonyms in addition to one preferred term for each class.

*SNOMED CT* is developed by the International Health Terminology Standard Development Organization (IHTSDO) [7]. It is the world’s largest clinical terminology and provides broad coverage of clinical medicine, including diseases and phenotypes. SNOMED CT includes pre-coordinated concepts (with their terms) and supports post-coordination, i.e., the principled creation of expressions (logical definitions) for new concepts. The U.S. edition of SNOMED CT dated March 2015 used in this work includes about 300,000 active concepts, of which 103,748 correspond to clinical findings.

*UMLS*. The Unified Medical Language System (UMLS) is a terminology integration system developed by the U.S. National Library of Medicine [8]. The UMLS Metathesaurus integrates many standard biomedical terminologies, including SNOMED CT. Although the version of UMLS available at the time of this investigation does not yet integrate HPO, it is expected to provide a reasonable coverage of phenotypes through its source vocabularies. In the UMLS Metathesaurus, synonymous terms from various sources are assigned the same concept unique identifier, creating a mapping among these source vocabularies. Terminology services provided by the UMLS support the lexical mapping of terms to UMLS concepts. We used the 2015AA version of the UMLS.

### Related work

#### Ontology matching

The general framework of this investigation is that of ontology matching. More specifically, we investigate different mappings techniques between the classes of two medical ontologies. Considering the matching techniques classification of Euzenat et al. [9], our approach falls under schema matching approaches, as it only relies on schema-level information. (Concepts in biomedical terminologies and ontologies represent classes, while the corresponding instances are found in EHR systems). Several techniques have been developed for schema matching and these approaches can be combined [10, 11]. Most relevant to our work are matching techniques that leverage the structural (i.e., the subsumption hierarchy of an ontology) and the lexical (i.e., the terms used as labels for the classes of an ontology) characteristics of the ontologies [12]. Establishing equivalence mappings is the most common approach to making two ontologies interoperable. However, partial mappings can advantageously extend interoperability when one ontology is finer-grained than the other [13].

Most ontology matching techniques have been developed for and applied to broad, ambiguous domains (e.g., the Semantic Web as a whole) and may not be as efficient when applied to specialized, less ambiguous domains, such as biomedicine. For example, when the ontologies to be matched cover different domains (e.g., DBpedia), bootstrapping the mappings with unsupervised filters to delimit the target domain can improve the quality of the resulting mappings [14]. However, while the improvement was significant for particularly ambiguous datasets, the domain filter did not improve (and could even decrease) the mapping quality for extremely specialized and unambiguous datasets, such as the subdomain “Pathological Function” in the UMLS [14]. Along the same lines, the BLOOMS system is an interesting solution for Linked Open Data (LOD) schema alignment, but has not been evaluated on LOD datasets from the life sciences domain [15].

In the next paragraphs, we review some relevant related work conducted in the in the medical domain on partial lexical mappings and partial logical mappings.

#### Partial lexical mappings

Particularly relevant to this investigation where we attempt to find partial lexical mappings for HPO concepts in SNOMED CT by removing some of modifiers that specialize phenotype terms in HPO is work done on the compositional aspects of biomedical terms. Terminologies, such as the Gene Ontology, have been shown to be highly compositional [16, 17] in that some of their more complex terms are derived from simpler terms by addition of modifiers. Moreover, it has been reported that the compositional structure of Gene Ontology terms impacts its usage [18] and can support automatic ontology extension [19]. Similarly, the compositional structure of SNOMED terms has been exploited for assessing the consistency of its hierarchical structure [20]. Recent work based on the compositionality of phenotype terms investigated skeletal abnormalities [21] and clinical phenotypes across species [22]. However in the latter study, the Entity-Quality decomposition strategy yielded better results on the Mammalian Phenotype Ontology than on HPO. Also of interest is the work involving partial mappings by Miličić *et al*. [23] in the context of mapping the rare diseases of the Orphanet terminology to the UMLS. Partial lexical mappings leveraging increasingly aggressive normalization of Orphanet terms were used to rank candidate mappings for comprehensive expert curation.

#### Partial logical mappings

We are not using supervised machine learning approaches in order to discover new partial mappings, as was done in [13]. Instead, we use existing equivalence relations between HPO and SNOMED CT and subsumption relations asserted in HPO to infer partial logical mappings. The resulting partial mappings denote a subclass relation between a fine-grained HPO concept and a more general SNOMED CT concept. A similar approach was used in a different domain to map adverse drug events (ADEs) between SNOMED CT and MedDRA. In this investigation, the fine-grained concepts in SNOMED CT were mapped to more general concepts in MedDRA through partial logical mappings [24].

### Specific contribution

The specific contribution of this work is not to propose new mapping techniques. Rather, we leverage existing techniques to extend the mapping of clinical phenotypes from HPO to SNOMED CT. More specifically, we leverage the lexico-syntactic properties of HPO terms and the logical structure of HPO to derive partial mappings. Moreover, we contrast the contribution of lexical and logical approaches to the development of partial mappings.

## Methods

Our investigation of partial mapping can be summarized as follows. We extracted phenotype concepts (along with their terms) from HPO and SNOMED CT. We identified complete lexical mappings between the two resources. We leveraged the lexico-syntactic properties of phenotype terms to derived partial lexical mappings, and the subsumption hierarchy of phenotype concepts to derive partial logical mappings. Finally, we analyzed the specific contribution of each approach and evaluated the quality of the partial mappings through manual review.

### Extracting phenotypes terms

From HPO, we selected the concept *Phenotypic abnormality* [HP:0000118] and all its descendants with their corresponding terms (preferred terms and synonyms). In order to restrict SNOMED CT to phenotypes and disorders, we selected the concept *Clinical Findings* [SCTID:404684003] and all its descendants, along with their terms (referred to as “descriptions” in SNOMED CT).

### Identifying complete lexical mappings

Although the focus of this investigation is on partial mappings, we rely on complete lexical mappings (denoting equivalence relations) for two reasons. Partial mappings are primarily useful for those concepts for which no complete mapping exists, and the complete lexical mappings are key to identifying partial logical mappings.

To identify equivalent mappings between HPO and SNOMED CT concepts, we mapped each original phenotype term (preferred term or synonym) from HPO to the clinical findings of SNOMED CT lexically through UMLS synonymy, as previously described in [3]. For example, the HPO concept *Abnormality of the mouth* [HP:0000153] has a complete lexical mapping to the SNOMED CT concept *Congenital anomaly of mouth* (*disorder*) [SCTID:128334002], as indicated by the UMLS Concept *Mouth Abnormalities* [C0026633] in which *Abnormality of the mouth* and *Congenital anomaly of mouth* (*disorder*) are synonyms. (The issue of congenitality will be addressed in the Discussion section.)

### Deriving partial lexical mappings

**Fig. 1:**
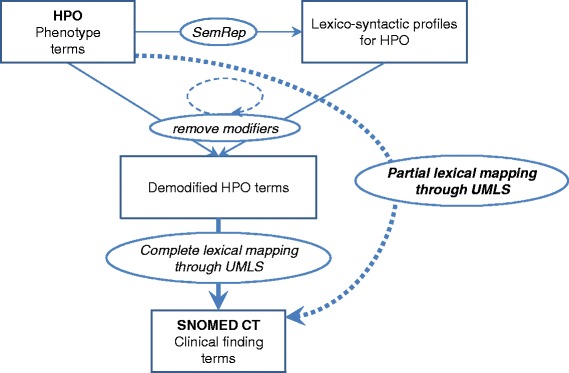
Identifying partial lexical mappings between HPO and SNOMED CT

To derive partial lexical mappings, we identified modifiers in phenotype terms (through lexico-syntactic analysis), and we performed increasingly aggressive demodification of HPO terms until the demodified HPO terms could be mapped to SNOMED CT (Fig. 1).

#### Identifying modifiers through lexico-syntactic analysis

In order to identify modifiers in HPO terms (preferred terms and synonyms), we performed a lexico-syntactic analysis (“shallow parsing”) of these terms using the minimal commitment parser available as part of natural language processing tool SemRep [25]. For example, the HPO term *Bilateral renal atrophy* [HP:0012586] is analyzed as two adjectival modifiers, *Bilateral* and *renal*, followed by the head noun *atrophy*. Its lexico-syntactic profile would therefore be recorded as [MOD-MOD-HEAD].

More specifically, we focused on terms with a [MOD]*[HEAD] profile (i.e., one or more adjectival or noun modifiers followed by a head noun). We also considered terms containing one prepositional attachment, in which we treated each element of the prepositional phrase as a modifier (of the main head noun) for the purpose of this analysis. Complex terms with multiple prepositional attachments were ignored, because their analysis requires more sophisticated parsing techniques.

#### Demodifying phenotype terms

Since our intuition is that modifiers in specialized HPO terms prevent mapping to the more general terms found in SNOMED CT, we attempted to remove the modifiers identified in HPO terms through lexico-syntactic analysis and to map the demodified terms to SNOMED CT through the UMLS, thereby creating a partial lexical mapping of the original HPO term to SNOMED CT. In practice, we iteratively removed all combinations of modifiers from an original HPO term (preferred term or synonym), in increasing order of aggressiveness, i.e., first removing one modifier at the time, then, two modifiers, etc. until only the head noun remained. For example, after removing the modifier *bilateral* from the HPO term *Bilateral renal atrophy* [HP:0012586], the demodified term *renal atrophy* mapped to SNOMED CT through the UMLS. Note that from this term, where the head noun *atrophy* is modified by *bilateral* and *renal*, we generated the following three demodified terms. By removing one modifier (“level-1”), we obtained *bilateral atrophy* and *renal atrophy*. After removing both modifiers (“level-2”), we generated *atrophy*. As an example of term with a prepositional attachment, *Congenital absence of uvula* [HP:0010292] has for lexico-syntactic profile [MOD HEAD][PREP HEAD]. Except for the head noun of the main noun phrase (*absence*), all the other lexical items are treated as modifiers (*congenital*, *of*, and *uvula*).

#### Mapping demodified terms through UMLS

We attempted a complete lexical mapping of the demodified HPO terms to SNOMED CT through the UMLS, as was done for the original HPO terms in [3]. Note that the complete mapping of a demodified term corresponds to the partial mapping of the original term prior to demodification. In order to select the closest mappings, we only recorded the mapping for the less demodified term(s). For example, there is no complete mapping to SNOMED CT for *Bilateral renal atrophy* [HP:0012586], but a “level-1” partial mapping is found to *Atrophy of kidney* [SCTID:197659005] after removing one modifier, *bilateral*.

### Deriving partial logical mappings

**Fig. 2:**
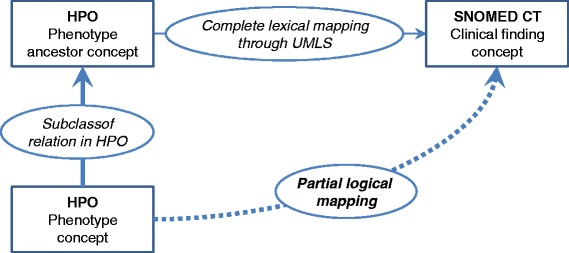
Identifying partial logical mappings between HPO and SNOMED CT

To derive partial logical mappings, we mapped HPO concepts to equivalent SNOMED CT concepts and we inferred partial logical mappings from the subsumption relations of HPO (Fig. 2).

Most HPO concepts have no complete lexical mapping (i.e., no equivalence relation) to SNOMED CT. For these concepts, we attempted a partial logical mapping. In practice, when an equivalent mapping to SNOMED CT was found among the ancestors of a given HPO concept, we inferred a partial logical mapping between this HPO concept and the SNOMED CT concept(s) equivalent to its ancestor. More specifically, if several ancestors of the HPO concepts have equivalence relations to SNOMED CT, we only record as partial logical mappings those ancestors that are the closest to the source HPO concept.

For example, the HPO concept *Oral cleft* [HP:0000202] has no complete lexical mapping in SNOMED CT. This concept is a subclass of *Abnormality of the mouth* [HP:0000153], which has an equivalent relation to the concept *Congenital anomaly of mouth* (*disorder*) [128334002] in SNOMED CT. Therefore, a partial logical mapping denoting a subclass relation is inferred between *Oral cleft* [HP:0000202] and *Congenital anomaly of mouth* (*disorder*) [128334002]. This logical mapping is deemed “level-1” because it is based on an equivalent mapping of a direct ancestor (i.e., parent concept). In the case of *Short upper lip* [HP:0000188], the resulting partial logical mapping was deemed “level-3” because its closest ancestor achieving a complete mapping was three levels above the source HPO concept (*Short upper lip* [HP:0000188] is a subclass of *Abnormality of upper lip* [HP:0000177], which is a subclass of *Abnormality of the lip* [HP:0000159], which is a subclass of *Abnormality of the mouth* [HP:0000153]).

### Evaluation

#### Quantitative evaluation

We quantified the number of complete lexical mappings and the number of partial mappings (lexical partial mappings and logical partial mappings) between HPO concepts and SNOMED CT concepts. The analysis was stratified by level of demodification for the partial lexical mappings and by level of subsumption for the partial logical mappings. Then we analyzed the overlap between partial lexical and logical mappings, as well as the combined coverage of HPO concepts provided by both types of partial mappings.

#### Qualitative evaluation

We evaluated the quality of the partial mappings by manual review of a random subset of 10 % of the partial lexical mappings. Additionally, we evaluated a sample of the partial logical mappings consisting of 25 mappings per level in the subsumption hierarchy. One of the authors (FD), a physician, tagged the partial mappings as ontologically valid if they were consistent with a subclass relation. For example, the mapping of *Bilateral renal atrophy* [HP:0012586] to *Atrophy of kidney* [SCTID:197659005] is ontologically valid. In contrast, the mapping of *Abnormality of the paranasal sinuses* [HP:0000245] to *Congenital malformation* (*disorder*) [SCTID:276654001] is not ontologically valid, because some subclasses of *Abnormality of the paranasal sinuses* (e.g., *Sinusitis* [HP:0000246]) are obviously not necessarily of congenital origin. (We will come back to this issue in the Discussion section).

Additionally, ontologically valid mappings were evaluated for clinical relevance from the perspective of cohort selection. In practice, the mappings were tagged as clinically relevant if they were “clinically useful” for building a cohort of patients exhibiting a particular phenotype, i.e., for selecting medical records describing the clinical phenotypes of such patients. For example, the mapping of *Bilateral renal atrophy* [HP:0012586] to *Atrophy of kidney* [SCTID:197659005] is deemed clinically useful, because it would be relatively easy to select patients with *Bilateral renal atrophy* from patients with *Atrophy of kidney*. In contrast, the mapping of *Abnormal respiratory motile cilium morphology* [HP:0005938] to *Morphologic finding* [SCTID:72724002] is not deemed clinically useful, because few patient records annotated with *Morphologic finding* would actually correspond to cases of *Abnormal respiratory motile cilium morphology*. In other words, this metric of clinical relevance attempts to assess whether the partial mappings are “close enough” for a specific use case, here cohort selection.

## Results

In this section, we present the results for each step of our approach to establishing partial lexical and logical mappings. We also provide an extended example to illustrate our mapping approach.

### Extracting phenotypes terms

From HPO, we selected 10,454 concepts specifically representing phenotypic abnormalities (10,454 preferred terms and 6158 synonyms). From SNOMED CT, we selected 103,748 concepts for clinical findings (103,748 fully specified names and 167,491 synonyms).

### Identifying complete lexical mappings

Of the 10,454 phenotype concepts in HPO, we identified a complete lexical mapping to clinical findings in SNOMED CT for (at least one term of the) 3096 HPO concepts (30 %). This proportion is consistent with our prior findings ([3]). We used the remaining 7358 concepts (10,631 terms) for identifying partial mappings lexically and logically.

### Deriving partial lexical mappings

#### Identifying modifiers through lexico-syntactic analysis

The lexico-syntactic analysis of the 10,631 HPO terms produced 494 distinct lexico-syntactic profiles, the most frequent of which being [MOD-HEAD] (23 %). The list of the 10 most frequent lexico-syntactic profiles (accounting for 65 % of the HPO terms) is shown in Table 1. A total of 6959 HPO terms had lexico-syntactic profiles amenable to demodification, corresponding to 35 distinct lexico-syntactic profiles. Of note, 218 HPO terms consisting of a single head noun ([HEAD]), were of course not amenable to demodification. The remaining 3454 HPO terms are complex terms and were not considered for demodification.

**Table 1:** Most frequent lexico-syntactic profiles of the 10,631 HPO terms not involved in a complete lexical mapping

Lexico-syntactic profile	Terms	(%)	Examples of HPO terms
[MOD—HEAD]	2478	(23 %)	*Oral cleft*, *Aplastic clavicles*, *Abnormal philtrum*
[MOD—MOD—HEAD]	1811	(17 %)	*Asymmetric limb shortening*, *Multicystic kidney dysplasia*
[HEAD] [PREP—DET—HEAD]	536	(5 %)	*Abnormality of the philtrum*, *Polydactyly of the foot*
[MOD—MOD—MOD—HEAD]	478	(4 %)	*Small proximal femoral epiphyses*, *Increased cup disc ratio*
[HEAD] [PREP—MOD—HEAD]	386	(4 %)	*Delay in motor development*, *Abnormality of renal excretion*
[MOD—HEAD] [PREP—HEAD]	321	(3 %)	*Hypertensive disorder of pregnancy*, *Coronal cleft of vertebrae*
[HEAD] [PREP—HEAD]	259	(2 %)	*Abnormality of upper lip*, *Tremor at rest*, *Tetralogy of Fallot*
[HEAD]	218	(2 %)	*Gastroschisis*, *Polydactyly*, *Pre*-*eclampsia*
[HEAD] [PREP—DET—MOD—HEAD]	209	(2 %)	*Abnormality of the paralabial region*, *Fragmentation of the metacarpal epiphyses*
[MOD—HEAD] [PREP—DET—HEAD]	202	(2 %)	*Downturned corners of the mouth*, *IgA deposition in the glomerulus*
top 10	6898	(65 %)	

A total of 2864 distinct modifiers extracted from these HPO terms were associated with 1838 distinct head nouns. The number of modifiers per term ranged from 1 to 8 (median = 2). The most frequent head nouns were *abnormality*, *hypoplasia*, *epiphyses*, *ossification*, *atrophy*, *phalanx*, *aplasia*, *phalanges*, *EEG* and *sclerosis*. Excluding prepositions, the most frequent modifiers were *abnormal*, *increased*, *absent*, *hypoplastic* and *decreased*.

#### Demodifying phenotype terms

The demodification process resulted in the creation of 23,936 demodified terms from the 6959 original terms.

#### Mapping demodified terms through UMLS

**Fig. 3:**
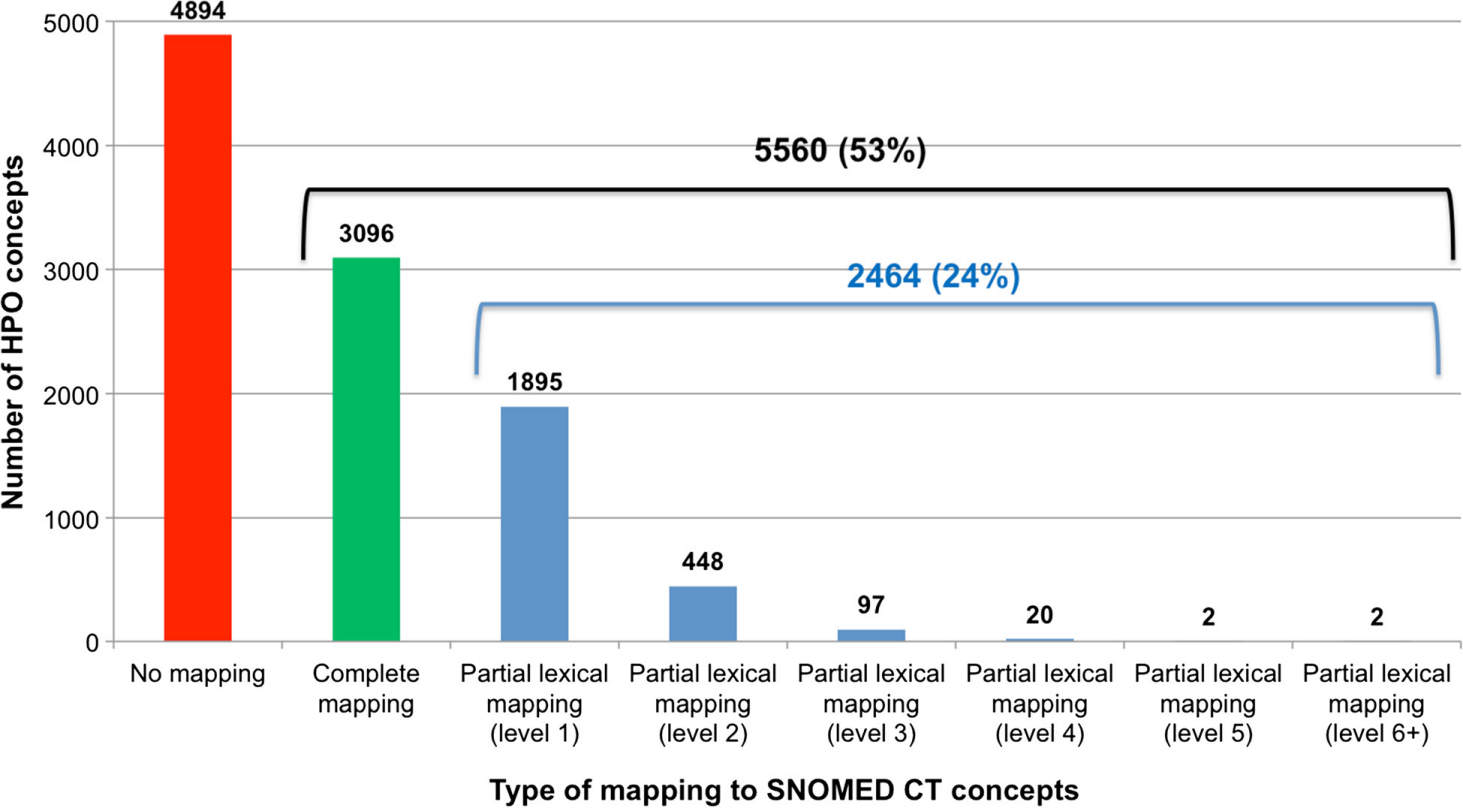
Complete and partial lexical mappings between HPO and SNOMED CT

Of the 7358 HPO concepts with no complete mapping to SNOMED CT, we identified a partial lexical mapping for (at least one term of the) 2464 HPO concepts (33 %). A majority of the partial mappings occurred at level 1 (i.e., after removing a single modifier). An analysis of the lowest level at which the mapping occurred is presented in Fig. 3. Among the modifiers, *metabolism*, *progressive*, *recurrent*, *generalized*, *abnormal*, *bilateral*, *morphology*, *distal*, *unilateral*, *epiphysis* and *congenital* are the most frequently removed when a mapping was found. The most frequent profiles involved in these mappings were [MOD-HEAD] (e.g., *Fasciculiform cataract* [HP:0010926]), [MOD-MOD-HEAD] (e.g., *Bilateral renal atrophy* [HP:0012586]), [HEAD][PREP-DET-HEAD] (e.g., *Osteosclerosis of the clavicle* [HP:0100923]), and [HEAD][PREP-MOD-HEAD] (e.g., *Abnormality of glutamine metabolism* [HP:0010903]).

### Deriving partial logical mappings

**Fig. 4:**
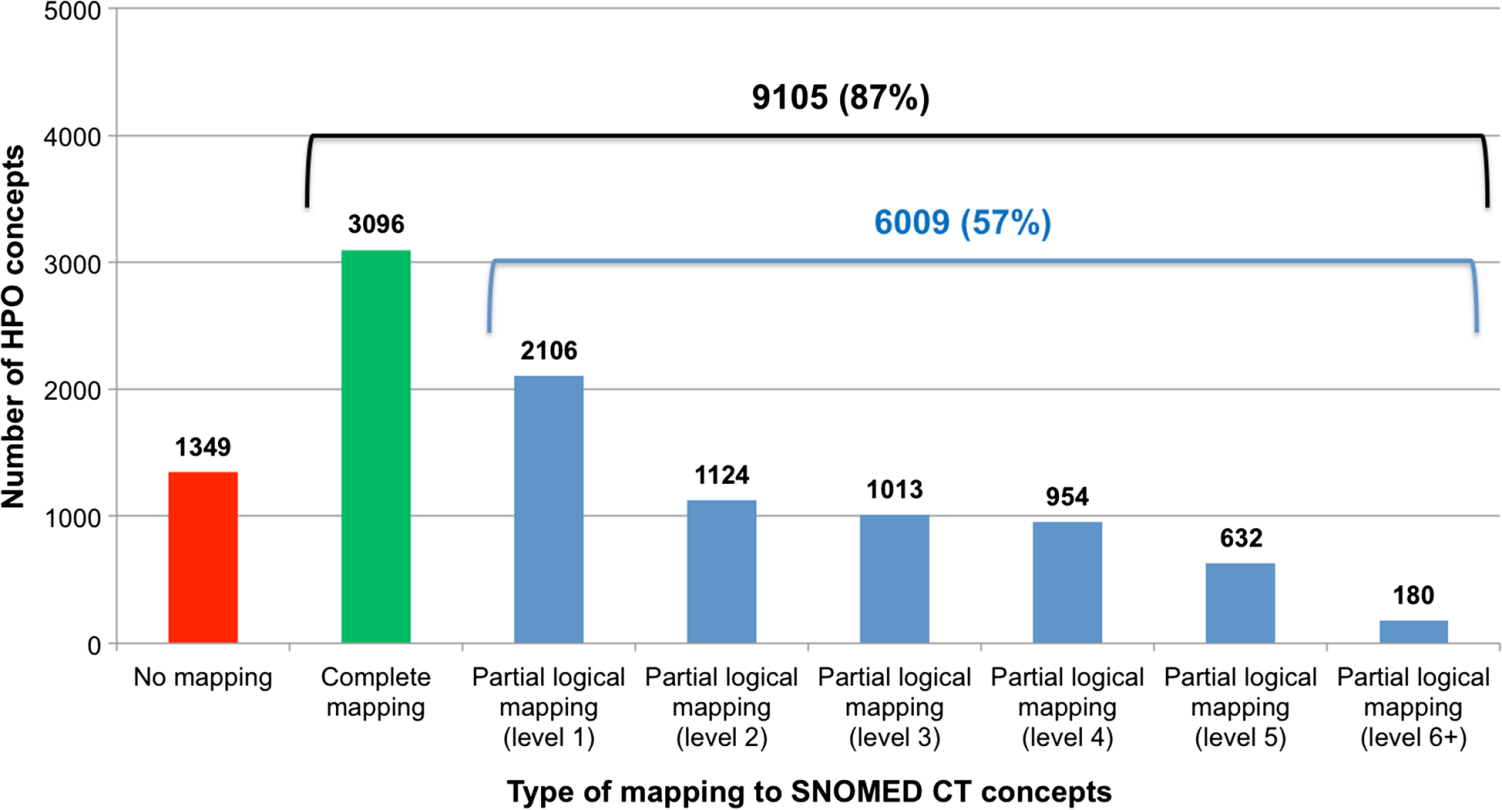
Complete and partial logical mappings between HPO and SNOMED CT

Of the 7358 HPO concepts with no complete mapping to SNOMED CT, we inferred a partial logical mapping for 6009 HPO concepts (82 %). The partial logical mappings were distributed across 10 levels of subsumption. The first level represented 2106 (35 %) of the partial logical mappings, and the first 4 levels represented 5197 (86 %) of all the partial logical mappings (Fig. 4).

### Evaluation

#### Quantitative evaluation

**Fig. 5:**
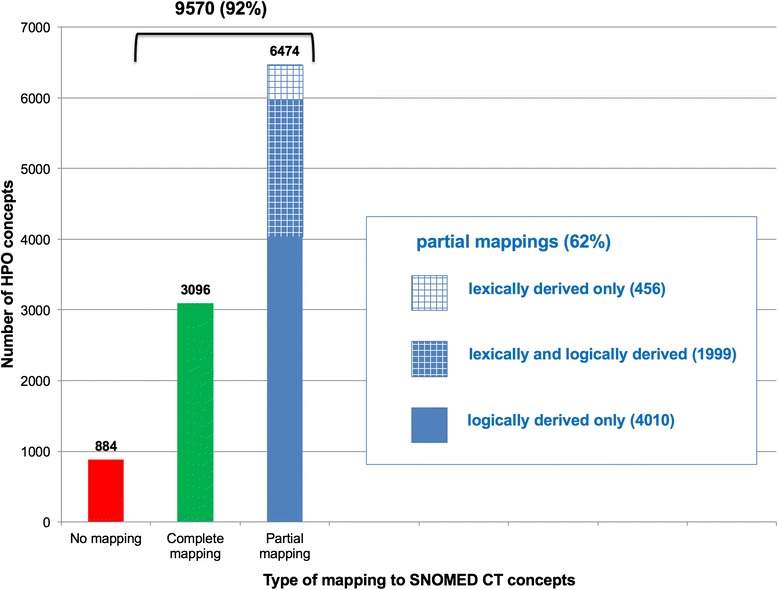
Partial logical mappings between HPO and SNOMED CT

Of the 10,454 phenotype concepts in HPO, we identified complete mappings for 3096 (30 %), partial lexical mappings for 2464 (24 %), and partial logical mappings for 6009 (57 %). As shown in Fig. 5, we identified partial mappings, lexical or logical, for 6474 HPO concepts (62 %).

#### Qualitative evaluation

In our randomly selected evaluation subset of 247 partial lexical mappings, 62 % were ontologically valid and 49 % were both ontologically valid and clinically relevant. As shown in Table 2, the quality of these mappings is higher for the first level of demodification.

**Table 2:** Qualitative evaluation of the partial lexical mappings

	ontologically valid mappings					clinically relevant mappings	
level	yes		no		total	(in proportion of the ontologically valid mappings)	
1	130	68 %	60	32 %	25	103	54 %
2	18	40 %	27	60 %	25	14	31 %
3+	5	42 %	7	58 %	25	4	33 %
all	153	62 %	94	38 %	125	121	49 %

Of the 125 logical mappings randomly selected among concepts with no lexical partial mappings, 71 % were ontologically valid and 67 % were both ontologically valid and clinically relevant. As shown in Table 3, the quality of the mappings is relatively consistent across the first 4 levels of logical mappings.

**Table 3:** Qualitative evaluation of the partial logical mappings, with no lexical mapping

	ontologically valid mappings					clinically relevant mappings	
level	yes		no		total	(in proportion of the ontologically valid mappings)	
1	22	88 %	3	12 %	25	20	80 %
2	19	76 %	6	24 %	25	17	68 %
3	15	60 %	10	40 %	25	15	60 %
4	18	72 %	7	28 %	25	17	68 %
5+	15	60 %	10	40 %	25	15	60 %
all	89	71 %	36	29 %	125	84	67 %

### Extended example

To illustrate the main steps of our partial mapping approach, we consider the HPO concept *Recurrent bronchitis* [HP:0002837], for which there is no complete lexical mapping to SNOMED CT.

#### Partial lexical mapping

The lexico-syntactic profile of this term is [MOD-HEAD], in which the head noun *bronchitis* is modified by the adjective *Recurrent*. We demodified this term by removing its sole modifier, *Recurrent*, resulting in the bare head noun, *bronchitis*. According to the UMLS, *bronchitis* is equivalent to three SNOMED CT concepts, *Bronchitis* (*disorder*) [SCTID:32398004], *Acute bronchitis* (*disorder*) [SCTID:10509002], and *Acute tracheobronchitis* (*disorder*) [SCTID:35301006]. Therefore, we identified a level-1 partial lexical mapping for *Recurrent bronchitis* [HP:0002837] to three target concepts in SNOMED CT.

#### Partial logical mapping

The concept *Recurrent bronchitis* [HP:0002837] has three direct ancestors in the subsumption hierarchy of HPO, *Abnormality of the bronchi* [HP:0002109], *Bronchitis* [HP:0012387] and *Recurrent upper respiratory tract infections* [HP:0002788]. According to the UMLS, the concept *Abnormality of the bronchi* [HP:0002109] has no equivalent in SNOMED CT. The concept *Bronchitis* [HP:0012387] is equivalent to the same three concepts identified as a mapping for the demodified term *bronchitis*. Finally, the concept *Recurrent upper respiratory tract infections* [HP:0002788] is equivalent to two SNOMED CT concepts: *Upper respiratory infection* (*disorder*) [SCTID:54150009] and *Recurrent upper respiratory tract infection* (*disorder*) [SCTID:195708003]. Therefore, we inferred a partial logical mapping for *Recurrent bronchitis* [HP:0002837] to five target SNOMED CT concepts, three from *Bronchitis* [HP:0012387] and two from *Recurrent upper respiratory tract infections* [HP:0002788]. Of note, since a partial mapping was found through a direct ancestor of *Recurrent bronchitis* [HP:0002837], we did not explore its more distant ancestors.

#### Overall

A partial mapping to SNOMED CT can be derived for the HPO concept *Recurrent bronchitis* [HP:0002837] both lexically and logically, at the first level (of demodification or subsumption) in both cases. Moreover, all the target concepts from the lexical mapping were also identified by the logical mapping, which also identified two additional target concepts.

## Discussion

### Enhanced mapping of phenotype concepts between HPO and SNOMED CT

In addition to the 30 % of HPO concepts that can be mapped to SNOMED CT through complete lexical mapping (through UMLS), we assessed that 62 % of all HPO concepts have a partial lexical or logical mapping to SNOMED CT, bringing to 92 % the proportion of HPO concepts mapped to SNOMED CT with an equivalent or subclass relation (Fig. 5). Partial mapping techniques significantly increase the rate of mapping for phenotype concepts between HPO and SNOMED CT, which confirms our intuition that HPO concepts tend to be more specialized than phenotype concepts in SNOMED CT, where they can often be mapped to more general phenotype concepts.

### Relative contribution of the partial lexical and logical mapping approaches

#### Overall

Unsurprisingly, the partial logical mapping approach is far more productive that the partial lexical mapping approach. More specifically, of the 7358 HPO concepts with no complete mapping to SNOMED CT, the proportion of partial mappings obtained is 82 % for the logical approach vs. 33 % for the lexical approach.

#### By level

Lexical and logical mappings also differ in the level at which the mapping occurs. A majority of the partial lexical mappings (95 %) occur after removing one or two modifiers (Fig. 3), while the partial logical mappings are distributed across a larger number of levels of subsumption (Fig. 4), with only 54 % of the mappings occurring over the first two levels. Although the levels for the lexical approach (i.e., number of modifiers removed) and for the logical approach (i.e., number of edges in the concept hierarchy) cannot be directly compared, this difference indicates that the lexical mappings are generally closer in meaning to the source HPO concept compared to the logical mappings.

#### Overlap between partial lexical and logical mappings

The overlap between the lexical and logical approaches to partial mapping is limited. As shown in Fig. 5, of the 6474 HPO concepts for which a partial mapping to SNOMED CT was identified, 1999 (31 %) were common to both approaches. In other words, the lexical approach only generated 456 mappings (7 %) that could not be derived logically.

For example, *Severe periodontitis* [HP:0000166] maps to *Periodontitis* (*disorder*) [SCTID:41565005] both lexically (at level 1) and logically (also at level 1). In contrast, *Vitamin B8 deficiency* [HP:0100506] maps to *Vitamin deficiency* (*disorder*) [SCTID:85670002] only through lexical mapping, and *Small face* [HP:0000274] maps to *Dysmorphic facies* (*finding*) [SCTID:248200007] only through logical mapping.

Of note, the “overlapping” partial mappings identified through lexical and logical approaches for a given source HPO concept are not always the same. For example, *Median cleft lip* [HP:0000161] maps to *Cleft lip* (*disorder*) [SCTID:80281008] lexically (at level 1) and to *Congenital anomaly of mouth* (*disorder*) [SCTID:128334002] logically (at level 3). As suggested by its closest proximity, the lexical mapping is more meaningful. One strategy for selecting between lexical and logical mappings for a given HPO concept when the mappings are different would be to give precedence to the mapping with the lowest level. A detailed comparison of the levels at which the mappings occur between the lexical and logical approaches is presented in Table 4.

**Table 4:** Comparison of the level of the partial mappings in the lexical and logical approaches

		Partial logical mapping						No logical	
		level 1	level 2	level 3	level 4	level 5	level ≥ 6	mapping	total
	level 1	1041	314	135	77	36	29	263	1895
Partial	level 2	137	55	58	23	9	3	163	448
lexical	level 3	13	20	8	17	5	0	34	97
mappings	level 4	4	6	2	3	0	0	5	20
	level 5	0	0	1	1	0	0	0	2
	level ≥ 6	0	0	0	2	0	0	0	2
No lexical mapping		911	729	809	831	582	148	884	4894
	total	2106	1124	1013	954	632	180	1349	7358

#### Qualitative aspects

As mentioned earlier, the quality of the partial logical mappings tends to be higher than that of the partial lexical mappings (71 % vs. 62 % for ontological validity and 67 % vs. 49 % for clinical relevance).

### Failure analysis

We investigated some of the cases where no partial mappings could be found and present the main reasons for failure.

#### Lexical partial mappings

Reasons for failure to derive a partial lexical mapping include terms with a head noun outside the domain of disorders, complex lexico-syntactic patterns not processed in this investigation, and complex lexical items identified as HEAD.

- Head noun outside the domain of disorders. For example, the HPO concept *Hypoplastic sacrum* [HP:0004590] is demodified to *sacrum*, for which cannot find a mapping to phenotypes in SNOMED CT, because *sacrum* is an anatomical entity. (In previous work, we have addressed this issue through the creation of post-coordinated expression [4].)
- Complex lexico-syntactic patterns. For example, *Complete duplication of the proximal phalanx of the 5th toe* [HP:0100415] has for lexico-syntactic pattern [MOD-HEAD][PREP-DET-MOD-HEAD][PREP-DET-MOD-HEAD]. We ignored noun phrases with multiple prepositional attachments from our processing and were therefore unable to identify a partial lexical mapping for this concept.
- Complex lexical items identified as HEAD. For example, *Pyruvate dehydrogenase complex deficiency* [HP:0002928] is a complex lexical item, which prevents it from being demodified.

#### Logical partial mappings

The main reasons for failure to derive a partial logical mapping is that none of the ancestors of the HPO source concept have an equivalent mapping to SNOMED CT through the UMLS. For example, none of the 10 ancestors of the HPO concept *Absent sternal ossification* [HP:0006628] has an equivalence to SNOMED CT. The limitations of the UMLS as a source of equivalence mappings between HPO and SNOMED CT directly impact our partial logical mapping approach, albeit in a relatively small way, since a partial logical mapping can be derived for 82 % of the HPO concepts (for which there is no equivalent mapping).

### Impact of implicit congenitality on the quality of the partial mappings

Congenitality tends to be expressed explicitly in SNOMED CT concepts, while it is often implicit in HPO concepts. For example, the HPO concept *Renal hypoplasia* [HP:0000089] is equivalent to *Congenital hypoplasia of kidney* (*disorder*) [SCTID:32659003] in SNOMED CT according to the UMLS. Here, congenitality is implied in HPO, because hypoplasia is always a congenital condition. In other cases, however, an HPO concept without mention of congenitality is mapped to a SNOMED CT concept with explicit mention of congenitality through the UMLS. For example, according to the UMLS, *Abnormality of the mouth* [HP:0000153] is equivalent to *Congenital anomaly of mouth* (*disorder*) [SCTID:128334002], which is not always true since not all mouth conditions occur congenitally. The conflation between congenital and non-congenital (or not-always-congenital) entities within the same UMLS concept can lead to incorrect partial mappings.

#### Partial lexical mappings

As mentioned earlier, the mapping of *Abnormality of the paranasal sinuses* [HP:0000245] to *Congenital malformation* (*disorder*) [SCTID:276654001] is inaccurate, because *Sinusitis* [HP:0000246], a subclass of *Abnormality of the paranasal sinuses*, is not necessarily of congenital origin. The problem here is the equivalence provided by the UMLS between *anomaly* and *Congenital malformation* (*disorder*) through the UMLS concept *Congenital Abnormality* [UMLS:C0000768].

#### Partial logical mappings

The mapping of *Abnormal calcification of the carpal bones* [HP:0009164] to *Congenital anomaly of the hand* (*disorder*) [SCTID:34111000] is inaccurate, because some calcifications can be acquired. The problem here is the equivalence provided by the UMLS between *Abnormality of the hand*, an ancestor of *Abnormal calcification of the carpal bones*, and *Congenital anomaly of the hand* (*disorder*) [SCTID:34111000] through the UMLS concept *Congenital Hand Deformities* [UMLS:C0018566].

#### Impact

The mapping of HPO concepts without mention of congenitality to SNOMED CT concepts with mention of congenitality is the main raison for creating partial logical mappings that are not ontologically valid. Since many HPO terms are demodified to the head noun *Abnormality* (mapped to *Congenital malformation*), this issue also has a profound impact on the quality of the partial lexical mappings. Furthermore, we estimated that the partial mappings would gain in clinical relevance (+11 % for partial lexical mappings and +2 % for partial logical mappings) if the issue of congenitality was addressed. This issue is of particular importance at a time when HPO intends to represent phenotypes not only for genetic diseases, but also for common diseases [26].

### Limitations and future work

One of the limitations of this work is that the mappings were investigated from the perspective of the source (HPO) rather than the target (SNOMED CT). More specifically, we report results in terms of proportion of the HPO concepts mapped to SNOMED CT without investigating the SNOMED CT concepts mapped to or the mappings themselves (i.e., the HPO-SNOMED CT concept pairs). Investigating the perspective of the target was beyond the scope of this work, but should be the object of future research.

Our partial lexical mapping approach only considers a limited number of lexico-syntactic profiles for the generation of demodified terms. Moreover, some of the lexical items characterized as HEAD by our shallow parser actually correspond to complex items, some of which could be amenable to demodification (e.g., *cortical cataract* from the HPO concept *Posterior cortical cataract* [HP:0010924] is identified as a single lexical item, but could be decomposed into the modifier *cortical* and the head noun *cataract*). However, further refinement of the lexical processes is unlikely to dramatically increase the performance of the partial lexical mapping approach.

The equivalence between HPO and SNOMED CT concepts derived through the UMLS is a key component of our partial logical approach. While SNOMED CT is fully integrated in the UMLS, HPO was not at the time of this investigation and we had to rely on the lexical tools provided by the UMLS to derive this mapping. HPO is now integrated in the UMLS (as of version 2015AB) and this curated mapping is likely to provide better equivalences between HPO and SNOMED CT concepts, which will be highly beneficial to our partial logical mapping approach.

## Conclusions

Through complete and partial mappings, 92 % of the 10,454 HPO concepts can be mapped to SNOMED CT (30 % complete and 62 % partial). Equivalence mappings between HPO and SNOMED CT allow for interoperability between data described using these two systems. However, due to differences in focus and granularity, equivalence is only possible for 30 % of HPO classes. In the remaining cases, partial mappings provide a next-best approach for traversing between the two systems. Both lexical and logical mapping techniques produce mappings that cannot be generated by the other technique, suggested that the two techniques are complementary to each other. The clinical relevance of the partial mappings (for a cohort selection use case) is 49 % for lexical mappings and 67 % for logical mappings. Finally, this work demonstrates interesting properties (both lexical and logical) of HPO and SNOMED CT and illustrates some limitations of mapping through UMLS.

## Abbreviations

HPO: Human Phenotype Ontology
UMLS: Unified Medical Language System
EHR: Electronic health records
LOD: Linked open data
